# Chitin Deacetylation Using Deep Eutectic Solvents: *Ab Initio*-Supported Process Optimization

**DOI:** 10.1021/acssuschemeng.0c08976

**Published:** 2021-03-05

**Authors:** Filipa A. Vicente, Matej Huš, Blaž Likozar, Uroš Novak

**Affiliations:** † Department of Catalysis and Chemical Reaction Engineering, National Institute of Chemistry, Hajdrihova ulica 19, 1000 Ljubljana, Slovenia; ‡ Association For Technical Culture of Slovenia (ZOTKS), Zaloška 65, 1000 Ljubljana, Slovenia

**Keywords:** chitin, deep eutectic solvents, greener deacetylation, density functional theory, deacetylation mechanism

## Abstract

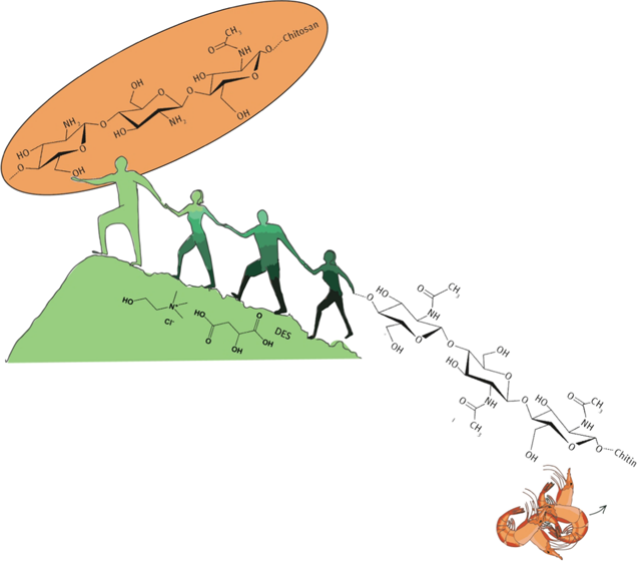

Chitin is the most abundant marine biopolymer, being recovered during the shell biorefining of crustacean shell waste. In its native form, chitin displays a poor reactivity and solubility in most solvents due to its extensive hydrogen bonding. This can be overcome by deacetylation. However, this process requires a high concentration of acids or bases at high temperatures, forming large amounts of toxic waste. Herein, we report on the first deacetylation with deep eutectic solvents (DESs) as an environmentally friendly alternative, requiring only mild reaction conditions. Biocompatible DESs are efficient in disturbing the native hydrogen-bonding network of chitin, readily dissolving it. First, quantum chemical calculations have been performed to evaluate the feasibility of different DESs to perform chitin deacetylation by studying their mechanism. Comparing these with the calculated barriers for garden-variety alkaline/acidic hydrolysis, which are known to proceed, prospective DESs were identified with barriers around 25 kcal·mol^–1^ or lower. Based on density functional theory results, an experimental screening of 10 distinct DESs for chitin deacetylation followed. The most promising DESs were identified as K_2_CO_3_:glycerol (K_2_CO_3_:G), choline chloride:acetic acid ([Ch]Cl:AA), and choline chloride:malic acid ([Ch]Cl:MA) and were subjected to further optimization with respect to the water content, process duration, and temperature. Ultimately, [Ch]Cl:MA showed the best results, yielding a degree of deacetylation (DDA) of 40% after 24 h of reaction at 120 °C, which falls slightly behind the threshold value (50%) for chitin to be considered chitosan. Further quantum chemical calculations were performed to elucidate the mechanism. Upon the removal of 40% *N*-acetyl groups from the chitin structure, its reactivity was considerably improved.

## Abstract 2

Chitin is the most abundant marine biopolymer, being recovered during the shell biorefining of crustacean shell waste. In its native form, chitin displays a poor reactivity and solubility in most solvents due to its extensive hydrogen bonding. This can be overcome by deacetylation.

## Introduction

Synthetic-based polymers have been produced from nonrenewable resources (petroleum and coal) since the World War II^1,2^ and are now getting accumulated in huge amounts in landfills, rivers, and oceans. These plastics are well-known for their hardness, flexibility, and resistance to most environmental phenomena. However, these qualities are also the drawbacks that lead to their persistence in the environment for very long periods of time, contaminating the soil and aqueous ecosystems.^1^ In 2018, 359 M tons of plastic were produced worldwide. Of these, 62 M tons were produced in Europe, of which only 29.1 M tons were collected to be treated.^3^ This clearly evidences a growing demand for environmentally friendly polymers. Therefore, the development and/or extraction of polymers derived from natural and renewable sources is of utmost importance as these are more easily degraded in nature.^4,5^

Chitin is the most abundant marine biopolymer in nature (annual growth of 100 billion tons). It is composed of repeating units of *N*-acetyl glucosamine (GlcNAc) connected by β-(1 → 4) linkages.^2,6−8^ It is mostly present in the exoskeleton of crustaceans and therefore recovered during the shell biorefining of crustacean waste (6–8 M tons produced/year).^6,7^ The use of this biowaste allows not only pollution reduction but also the recovery of high-added-value products, namely, proteins (20–40%), calcium carbonate (20–50%), chitin (15–40%), and several minor components including lipids, astaxanthin, and other minerals,^6,7,9^ and contributes to the circular economy approach for a greener future.^9,10^

In its native form, chitin displays poor reactivity and solubility in most solvents due to its extensive hydrogen-bonding network, necessitating a conversion to its water-soluble derivative—chitosan, which is suitable for a wider range of applications.^5,7,8,11^ This is accomplished by deacetylation. At least a 50% degree of deacetylation (DDA) is required. Currently, there are two distinct approaches to obtain chitosan, namely, through chemical and biological processes.^12^ The first requires harsh conditions, for instance, high concentrations of NaOH (≥40 wt %) and high temperatures (≥100 °C) for several hours or days, whereas the latter process uses enzymes.^11^ Even though biological methods comprise a more sustainable approach, they are not industrially employed owing to the high costs of enzymes and regulatory concerns.^11,12^ As a result, a cheaper chemical approach is used despite producing large amounts of toxic and corrosive wastewater. Recently, a few authors have proposed the reduction of NaOH employed by decreasing the solid/liquid ratio from the typical 1:50 to 1:10^13^ or 1:5^14^ upon the mechanochemical conversion of chitin into chitosan. However, this leads to the formation of low-molecular-weight chitosan^13^ or requires an aging process of up to 6 days with a 98% relative humidity with saturated aqueous solutions of K_2_SO_4_.^14^ Therefore, it is imperative that a more biocompatible and cost-effective platform for chitin deacetylation be developed. An attractive alternative is the use of deep eutectic solvents (DESs) as these greener solvents have already demonstrated outstanding performances for chitin extraction,^15−18^ dissolution,^19,20^ processing,^21^ and formation of films^22^ and nanofibers.^23^

DESs are mixtures of pure compounds, usually a hydrogen-bond acceptor (HBA) and a hydrogen-bond donor (HBD), for which the eutectic point temperature is below that of an ideal liquid mixture.^24^ As analogous solvents to ionic liquids, DESs share most of their unique properties but usually present a greener character and cheaper and easier preparation. Therefore, DESs have been applied over the last few years as alternative solvents and/or catalysts for organic transformations in general, polymerization reactions, biomass processing, and separation processes.^25^ In this manner, DESs emerged as a promising alternative to the conventional and hazardous approach used during the chemical deacetylation of chitin. Thus, this work aimed at reporting for the first time the possibility of performing chitin deacetylation using these environmental-friendly solvents as well as the mechanisms behind this process through quantum chemical calculations.

## Experimental Section

### Materials

Four HBAs were used in this work, namely, choline chloride ([Ch]Cl, ≥98% purity) and choline dihydrogen citrate ([Ch]DHC, 99% purity) from Sigma-Aldrich, potassium bicarbonate (99.7% purity) from Honeywell Fluka, and potassium carbonate (99% purity) from Merck. Regarding the HBDs, glycerol (G, 98–101% purity) acquired from Pharmachem Sušnik, ethylene glycol (EG, ≥99.5% purity) purchased from Fluka, acetic acid glacial (AA, 100% purity) supplied by Honeywell, and oxalic acid (OA, 98% purity), malic acid (MA, 98% purity), and citric acid monohydrate (CA, 100.5% purity) from Sigma-Aldrich were used. Sodium hydroxide (98% purity) was obtained from Honeywell Fluka. Chitin from shrimp shells (practical grade, powder) was acquired from Sigma-Aldrich.

### Methods

#### Quantum Chemical Calculations

For electronic structure calculations, Gaussian 16 was used.^26^ Quantum chemical calculations were performed using the LCAO method and the density functional theory. A hybrid functional (M06-2X)^27^ with Pople’s basis set 6-311++g(d,p)^28−31^ sufficed for well-converged results. M06-2X is known to predict accurately the main group thermochemistry and to account for dispersion interactions, which are neglected by vanilla DFT.^32^

Intermediates and transition states were relaxed until the forces acting on all atoms dropped below 1.5 × 10^–5^ hartree/bohr and checked with vibrational analysis to confirm that they had zero or one imaginary frequency, respectively. The transition states were identified using the synchronous transit-guided quasi-Newton method (STQN) and verified by following the intrinsic reaction coordinate (IRC). Vibrational analysis was used to obtain the necessary parameters for the calculation of the translational, rotational, and vibrational parts of the partition function, from which the enthalpic and entropic contributions to the Gibbs free energies at 298 K and 1 atm were calculated as in ref (33).

As the model compound for studying deacetylation, GlcNAc was chosen. The mechanism for the cleavage of its amide bond with various reactants was studied: OH^–^ and H_3_O^+^ (conventional alkaline and acidic hydrolysis in aqueous solutions), glycerol, bicarbonate, acetic acid, oxalic acid, malic acid, choline chloride, K_2_CO_3_:G, [Ch]Cl:AA, [Ch]Cl:OA, and [Ch]Cl:MA. Implicit solvation as implemented in the SMD variation of IEFPCM was used.^34^

#### DES Preparation

Each HBD was mixed with all of the HBAs under study at 80 °C, in the molar ratio depicted in Table 1, while considering the amount of water present in each compound. The water content was previously measured using a Metrohm 831 Karl Fischer coulometer for the liquid compounds and a moisture analyzer HE53 from Mettler Toledo for solid compounds. The compounds were mixed until a homogeneous and clear liquid was formed, resulting in the formation of several DESs.

**Table 1:**
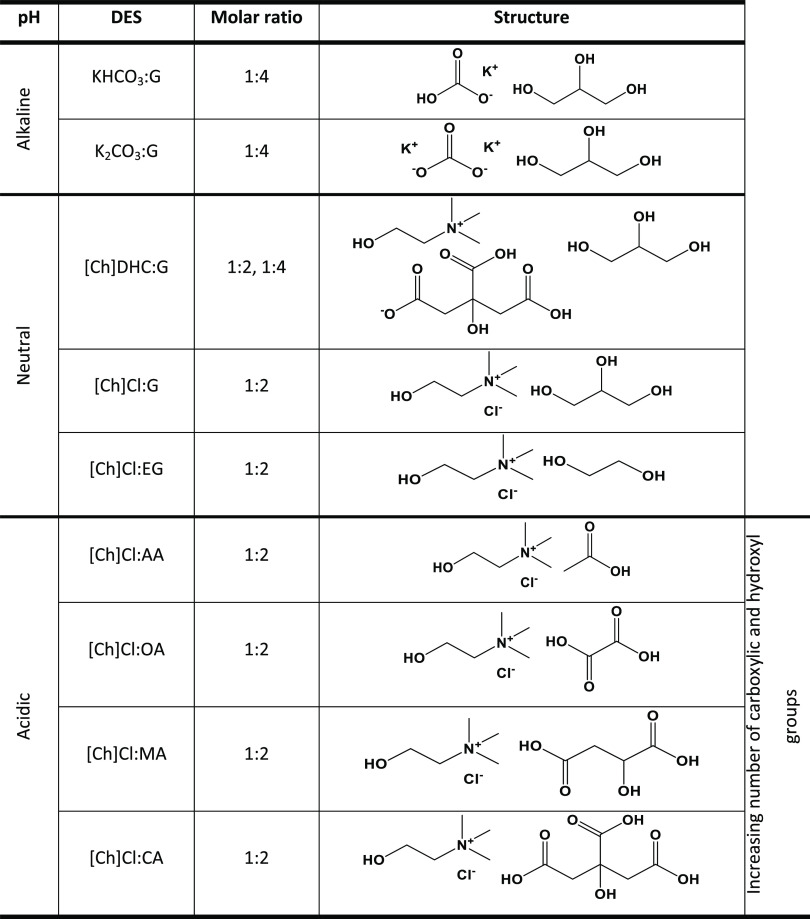
DESs Studied in This Work and Their Respective Molar Ratios and Structure

#### Chitin Deacetylation

Chitin deacetylation was carried out by mixing the solvent with chitin in a solid/liquid ratio of 1:50 (w/V) at 80 °C for 24 h. To stop the reaction, distilled water was added to the mixture, allowing the precipitation of chitin/chitosan that was later filtrated and washed multiple times. The solid portion was dried overnight at 35 °C and analyzed through attenuated total reflection-Fourier transform infrared (ATR-FTIR) spectroscopy. The same procedure was applied for the hydrotropy as well as the temperature (80, 100, and 120 °C) and time (2–24 h) studies.

#### ATR-FTIR Spectroscopy

All analyses were performed at room temperature with a Spectrum two (PerkinElmer, Manchester, U.K.) in the range of 4000–650 cm^–1^ by accumulating 32 scans with a resolution of 4 cm^–1^ and an interval of 2 cm^–1^. FTIR spectra of commercial chitin and chitosan are presented in Figure S1. The DDA of each sample was determined by the correlation between the absorption bands at 1320 cm^–1^ (amide III band) and 1420 cm^–1^ (reference band) as proposed in eq 1 by Brugnerotto and co-workers.^35^
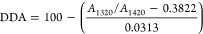
1

#### X-ray Diffraction

Commercial chitin and chitosan as well as the samples from the most promising DESs and from NaOH were characterized by powder XRD and recorded on a PW3040/60 X’Pert PRO MPD diffractometer, which was operated at 45 kV and 40 mA with a Cu Kα radiation source (λ = 0.154056 nm) at room temperature with a step size of 2° in the 2θ range from 5 to 40°. The crystalline index (CrI, %) was determined according to eq 2.
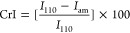
2where *I*_110_ is the maximum intensity of the (110) diffraction peak at 2θ = 20° and *I*_am_ is that of the amorphous diffraction signal at 2θ = 16°.

## Results and Discussion

Chitin displays a rigid structure, making it very difficult for the solvent to penetrate and facilitate its dissolution and deacetylation. Nevertheless, DESs have shown promising results in chitin dissolution and allow an easier diffusion of the solvent inside the biopolymer structure.^20^ Therefore, we chose these neoteric solvents for possible simultaneous dissolution and deacetylation of chitin, considering that it is possible to engineer the DESs based on the target application. We stress that the capacity for deacetylation is contingent on the ability to dissolve chitin beforehand. Herein, we started by calculating the barriers of the proposed mechanism for conventional deacetylation of chitin by means of DFT calculations, which then served as a benchmark for the comparison to the calculated barriers for the same reaction with the studied DESs. Afterward, the same protocol was followed for a few DESs, namely, some of the most common acidic DESs ([Ch]Cl:AA, [Ch]Cl:OA, and [Ch]Cl:MA),^36^ the well-known alkaline DES (K_2_CO_3_:G),^37^ and a common neutral DES ([Ch]Cl:G).^36^ These DESs were selected considering that the literature suggests that only strong acids and bases are capable of performing chitin deacetylation. Lastly, the experimental approach was carried out to truly evaluate the DES’s capacity to perform this deacetylation.

### Molecular Modeling

#### Alkaline and Acidic Hydrolysis in Aqueous Solutions

First, quantum chemical calculations were performed to understand the mechanism of the conventional (basic and acidic) deacetylation process.

Amides undergo hydrolysis in acidic or alkaline solutions via a well-known mechanism.^38−40^ In alkaline conditions, OH^–^ attacks the carbonyl carbon, yielding a tetrahedral intermediate, which then ejects the amine. In acidic conditions, amidic oxygen is first protonated, activating the adjacent carbon atom for the attack with water. The ensuing intermediate then expels the amine. If DESs are desired to be able to deacetylate, then the mechanism must have comparable reaction barriers. We therefore compare the calculated activation barriers and reaction energies for the reaction with DESs to those of alkaline and acidic hydrolysis in water.

**Figure 1:**
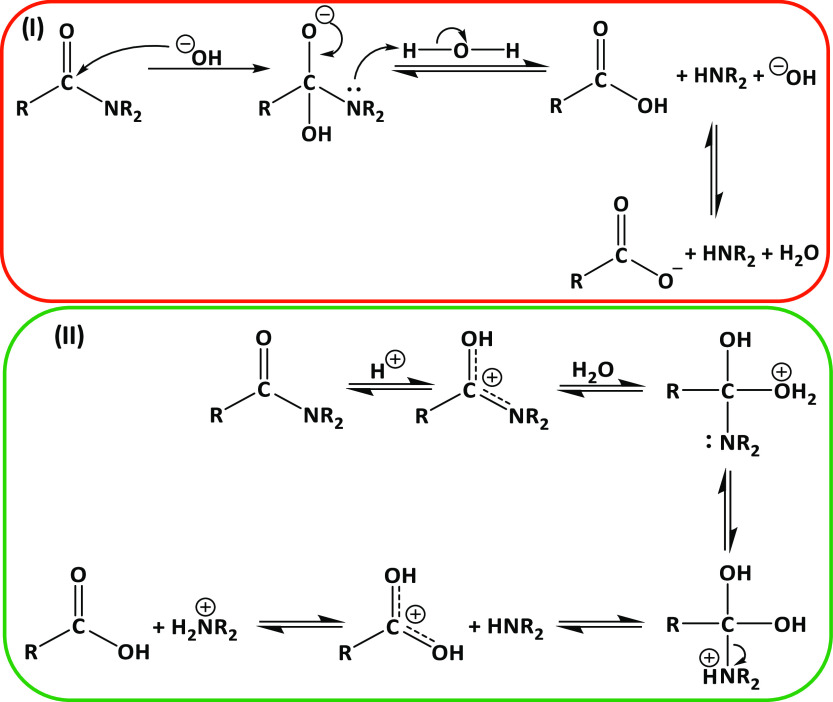
Mechanism of amide hydrolysis in alkaline (I) and acidic (II) aqueous solutions.

As shown in Figure 1, alkaline hydrolysis can be modeled as a two-step process.^38,39^ First, the attacking OH^–^ binds to the amidic carbon, which undergoes a C–N scission in the subsequent step. The calculated Gibbs free energy barriers for these steps are 22 and 21 kcal·mol^–1^, respectively. The overall reaction is exothermic (Δ*G* = −7 kcal·mol^–1^). These values are consistent with Car–Parrinello molecular dynamics simulations from Zahn, where the barriers for H^+^- and OH^–^-assisted hydrolysis were calculated to be 19 and 16 kcal·mol^–1^, respectively.^41,42^

In an acidic environment, protonation of amide is a fast first-step.^38,39^ The rate-determining step is the addition of the water molecule to a protonated amide, which has a calculated barrier of 27 kcal·mol^–1^. As the solvent actively participates in this step—OH^–^ originates from one water molecule, while H^+^ originates from another one—an additional water molecule was accounted for in the vicinity of the active site. Neglecting this effect, which in effect means that both OH^–^ and H^+^ would originate from the same water molecule, yields a higher calculated barrier of 41 kcal·mol^–1^. This would be the case in the gaseous phase. Both transition states are shown in the SI (Figure S2). A subsequent C–N cleavage is fast with a negligible barrier of 3 kcal·mol^–1^. Again, the overall reaction is slightly exothermic (Δ*G* = −5 kcal·mol^–1^).

In aqueous solutions of carbonate, which are alkaline, the bicarbonate ion (HCO_3_^–^) is also present. However, the first step in the reaction is prohibitively slow (38 kcal·mol^–1^) for the reaction to be feasible. In this step, the bicarbonate ion would bind to the amidic carbon through one of its oxygen atoms. In a subsequent fast step with a barrier of 7 kcal·mol^–1^, the hydrogen atom would migrate to the nitrogen atom, thereby also cleaving the C–N bond. The reaction is also endothermic. However, the barrier for the reverse reaction (decomposition of the bicarbonate-amide intermediate) is only 3 kcal·mol^–1^, meaning that the intermediate is unstable and that it would readily decompose; the cumulative barrier must be viewed as an apparent barrier in this case, which is also larger than in the case of OH^–^. This does not mean that in aqueous carbonate solutions, deacetylation is not possible; it means that the principal active species is OH^–^ rather than HCO_3_^–^. One should not overinterpret these values and rather use them with caution. The calculated activation barriers, valid only within the approximations made, unequivocally show which reaction is the most temperature-dependent, while the reaction rate is *also* a function of the pre-exponential factors.

**Figure 2:**
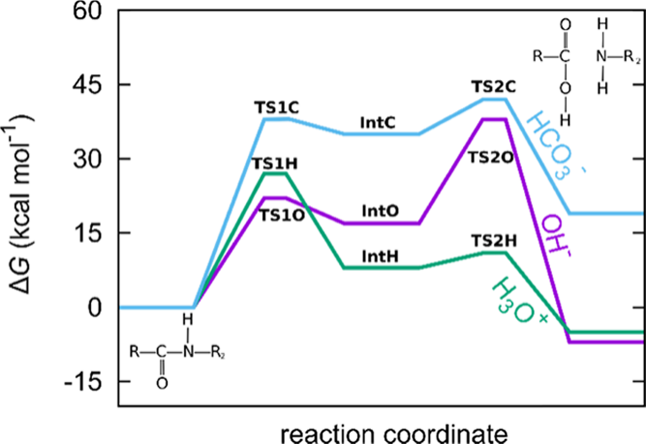
Calculated Gibbs free energies for amide hydrolysis with H_3_O^+^, OH^–^, and HCO_3_^–^. See Figure S3 for structures.

In Figure 2, the relevant structures and the calculated Gibbs free energies for all three mechanisms are drawn.

### Chitin Deacetylation

#### Computational Screening

Since we know that amide hydrolysis, in particular chitin deacetylation, readily proceeds in strongly alkaline or acidic conditions, the above-calculated barriers provide a benchmark. DESs exhibiting barriers around 20–30 kcal·mol^–1^ are therefore labeled as promising. See Table 2 for a summary and Figure S4 for all of the calculated Gibbs free energies.

**Table 2:** Comparison of the Calculated Rate-Determining Barriers for the Deacetylation of GlcNAc with Different DESs, and OH^–^ and H_3_O^+^ for Reference

DES formulation	rate-determining barrier (kcal·mol^–1^)
K_2_CO_3_:G	26
[Ch]Cl:G	31
[Ch]Cl:AA	23
[Ch]Cl:OA	25
[Ch]Cl:MA	19
OH^–^	22
H_3_O^+^	27

The rate-determining barriers listed in Table 2 warrant a closer inspection. Low values imply that a certain formulation is a feasible deacetylation agent and allow for the comparison of analogous mechanisms (for instance, among [Ch]Cl:AA, [Ch]Cl:OA, and [Ch]Cl:MA). However, this does *not* mean that [Ch]Cl:MA (*E*_A_ = 19 kcal·mol^–1^) is a better deacetylation agent than strongly alkaline aqueous solutions (*E*_A_ = 22 kcal·mol^–1^). While lower values in general mean that the reaction tends to be faster, other factors also affect the reaction rates. For instance, transition states involving DESs are termolecular (including three molecules), while those involving OH^–^ or H_3_O^+^ are bimolecular. Termolecular reactions are slower due to steric hindrance.

##### K_2_CO_3_:G

In this nonaqueous mixture, the carbonate ion and glycerol molecule can be active species. The carbonate ion first binds to the amidic carbon atom through an oxygen, which has a barrier of 26 kcal·mol^–1^. A subsequent C–N bond cleavage, mediated by glycerol, has a barrier of 8 kcal·mol^–1^. The reaction is thus feasible.

##### [Ch]Cl:G

The reaction proceeds in a two-step fashion. First, the glycerol molecule through its hydroxyl oxygen attaches to the amidic carbon atom, while the hydroxyl proton binds to the amidic oxygen atom, which has a reaction barrier of 31 kcal·mol^–1^. In a subsequent step, the proton migrates to the amidic nitrogen, cleaving the C–N bond in the process, which has an activation barrier of 27 kcal·mol^–1^. A one-step mechanism, where the C–N cleavage is concerted with the attack of glycerol, has a higher barrier of 40 kcal·mol^–1^. The choline cation does not participate in the reaction but instead only allows for the formation of DES.

##### [Ch]Cl:AA

**Figure 3:**
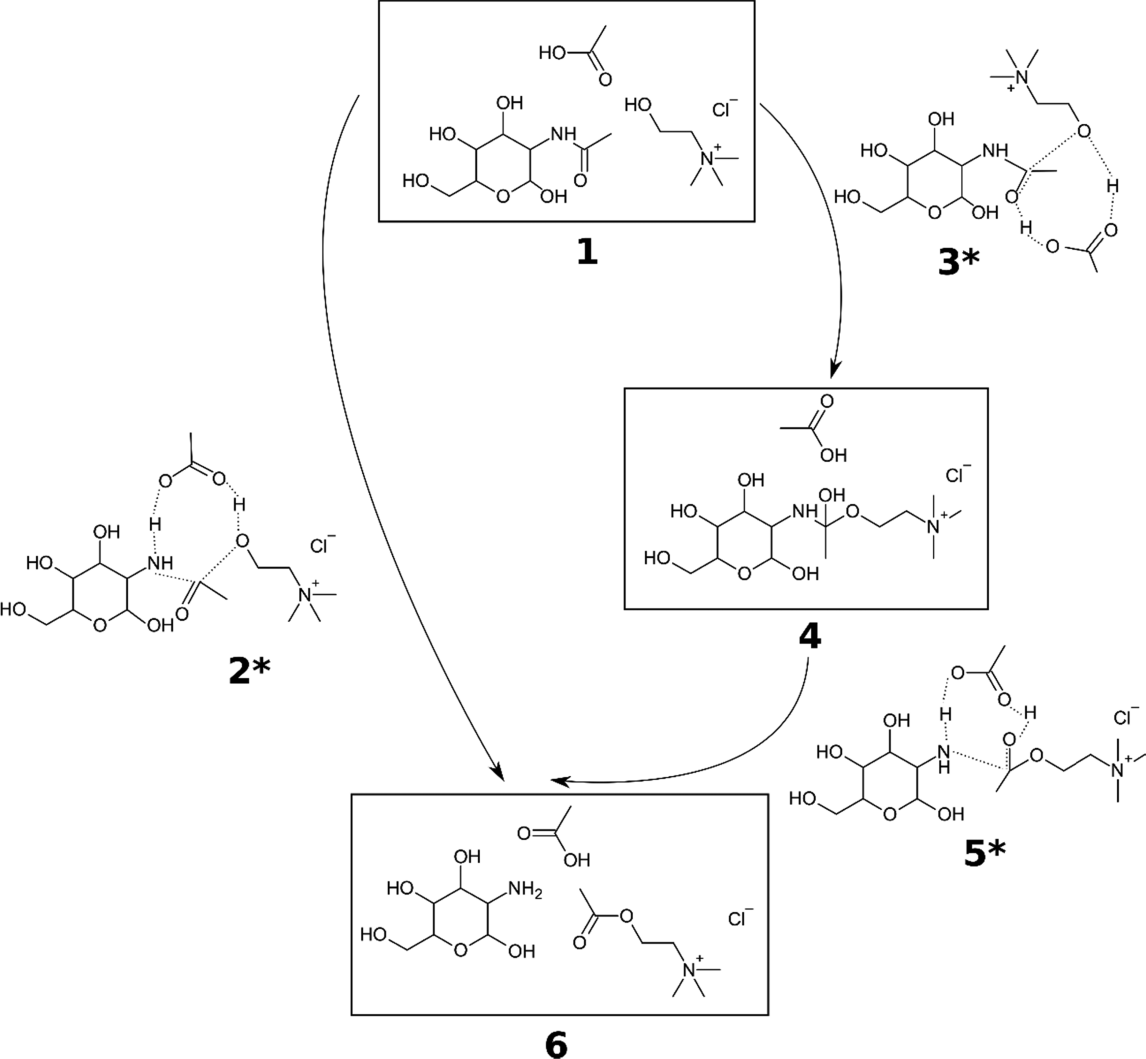
Structures (intermediates and transition states, marked with asterisks) in deacetylation of GlcNAc with [Ch]Cl:AA. The reaction can proceed as a one-step (1,2*,6) or a two-step transformation (1,3*,4,5*,6). With [Ch]Cl:OA and [Ch]Cl:MA, the mechanism is analogous. For the exact geometry, see Figure S5.

Possible active species are the choline cation and acetic acid (Structure **1** in Figure 3), which can react in a concerted or two-step fashion. When they cooperate concertedly (2*), the oxygen atom from choline binds to the amidic carbon atom, while acetic acid protonates the nitrogen atom, simultaneously causing the C–N bond scission. The barrier for the concerted mechanism is 32 kcal·mol^–1^, which should be attainable. In a two-step mechanism, the same C–O bond is formed (3*), but the acid protonates the amidic oxygen instead (barrier of 26 kcal·mol^–1^) (**4**). In the next fast step with a barrier of 2 kcal·mol^–1^ (5*), the C–N bond is broken via a proton migration (**6**). Due to similar activation barriers, the pathways coexist.

##### [Ch]Cl:OA

Analogous mechanisms (concerted *or* two-step) exist as for [Ch]Cl:AA. The barrier for the concerted mechanism is 25 kcal·mol^–1^, while the barriers for the two-step mechanism are 44 kcal and 4 kcal·mol^–1^. This hints that this mixture is also suitable for deacetylation.

##### [Ch]Cl:MA

This mixture also follows an analogous mechanism. The barrier in a concerted mechanism is 19 kcal·mol^–1^, while for the two-step mechanism the rate-determining barrier is 22 kcal·mol^–1^. This renders [Ch]Cl:MA the most promising of the screened DES formulations.

#### Experimental Screening

Herein, different DES were prepared using four HBAs: potassium bicarbonate (KHCO_3_), potassium carbonate (K_2_CO_3_), choline dihydrogen citrate ([Ch]DHC), and choline chloride ([Ch]Cl); and six HBDs: glycerol (G), ethylene glycol (EG), acetic acid (AA), oxalic acid (OA), malic acid (MA), and citric acid (CA). In general, the molar ratio HBA/HBD was chosen as 1:2, unless a different ratio was required due to solubility (miscibility) or performance issues. For instance, for K_2_CO_3_, it is only possible to form a DES with a molar ratio of at least 1:3.5;^37^ therefore, a 1:4 ratio was used in this work. The same ratio was chosen for KHCO_3_ for comparison purposes. Moreover, as [Ch]DHC:G was used to study the influence of the molar ratio on chitin deacetylation, it was prepared at both (1:2 and 1:4) molar ratios.

**Figure 4:**
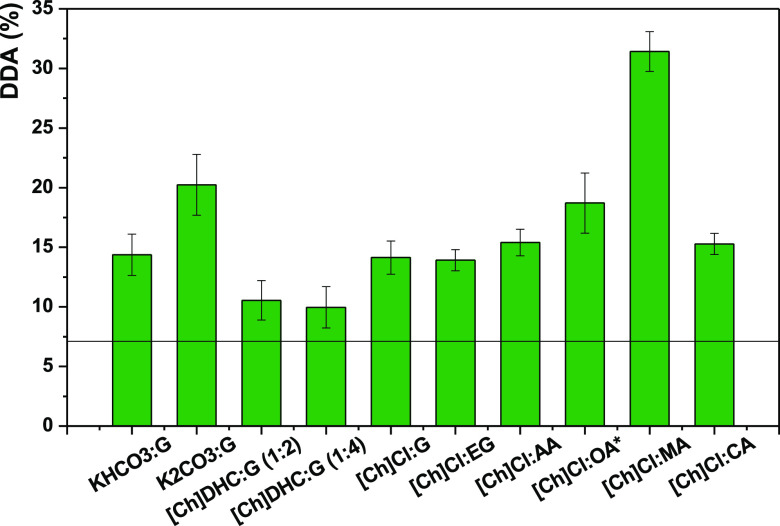
Performance of different DESs (pure solvents) on chitin deacetylation at 80 °C for 24 h. The line represents the DDA of commercial chitin (7 ± 1%). The * indicates that in the particular case of [Ch]Cl:OA, the deacetylation was performed at 80 °C but for 4 h.

Once formed, DESs were tested for their ability to promote chitin deacetylation, as described in the Methods section. Their performance was evaluated by their DDA, and the results are compared with the initial DDA of chitin (7 ± 1%). The results are summarized in Figure 4, and the respective FTIR spectra are shown in Figure S6. As seen, DES can be divided into three groups: (i) those effecting negligible deacetylation (such as [Ch]DHC:G); (ii) those with modest deacetylation (DDA around 15%), such as [Ch]Cl:EG ≈ [Ch]Cl:G < KHCO_3_ < [Ch]Cl:CA < [Ch]Cl:AA; and (iii) DES with a high deacetylation potential (DDA above 20%), specifically [Ch]Cl:OA < K_2_CO_3_:G < [Ch]Cl:MA. We also stress that after deacetylation with [Ch]Cl:OA at 80 °C for 24 h, the sample changed from a whitish powder to black, indicating a full degradation of the polymer as opposed to controlled deacetylation. In fact, it has been previously reported that this DES is too acidic and sometimes leads to the degradation of carbohydrates.^43^ Hence, the deacetylation with [Ch]Cl:OA was followed in a shorter timespan: between 2 and 8 h, as shown in Figure S7. The deacetylation was the most efficient after 4 h, achieving a DDA of 19 ± 3%, which is plotted in Figure 4.

This is consistent with DFT screening, where [Ch]Cl with acids (MA, OA, AA) was identified as having suitably low barriers for deacetylation to be feasible.

Due to physical difficulties with the final product, which render this DES industrially unsuitable, [Ch]Cl:OA was not studied further despite the encouraging initial performance. As seen in Figure 4, the trend in deacetylation for the acidic-based DES (measured with ascending DDA) is [Ch]Cl:AA < [Ch]Cl:OA < [Ch]Cl:MA, which roughly follows the number of hydroxyl and carboxylic groups in the acid. This increases the polar character of the acids and increases the number of hydrogen bonds they can form, possibly allowing a larger perturbation of the hydrogen bonding in native chitin.

The exception is [Ch]Cl:CA, which is, despite being a tricarboxylic acid, the bulkiest acidic DES, suffering from a steric effect preventing it from diffusing noticeably into the rigid chitin structure and perturbing it. Consequently, this DES presents the lowest DDA within the acidic-based DESs.

K_2_CO_3_:G also showed interesting results, promoting a DDA of ∼20% without any optimization. This is due to the fact that it acts as a strong base and follows a conventional alkaline hydrolysis mechanism (vide supra).

Upon a proper selection of DES, it is possible to promote chitin deacetylation to some degree. We now focus on improving yields and explaining the mechanism.

#### Hydrotropy Study

It is well-known that ionic liquids display a hydrotropic mechanism when solvating more hydrophobic compounds.^44^ More precisely, aqueous solutions of hydrotropes (water-soluble compounds characterized by an amphiphilic structure, yet unable to form micelles),^45^ such as ionic liquids, are able to increase the solubility of water-insoluble or sparingly water-soluble organic compounds due to the formation of aggregates.^44,46^ Interestingly, DESs have recently been shown to also be able to act as hydrotropes,^46,47^ leading to an enhanced solubility of the lignin’s monomers as well as different types of lignins themselves.

**Figure 5:**
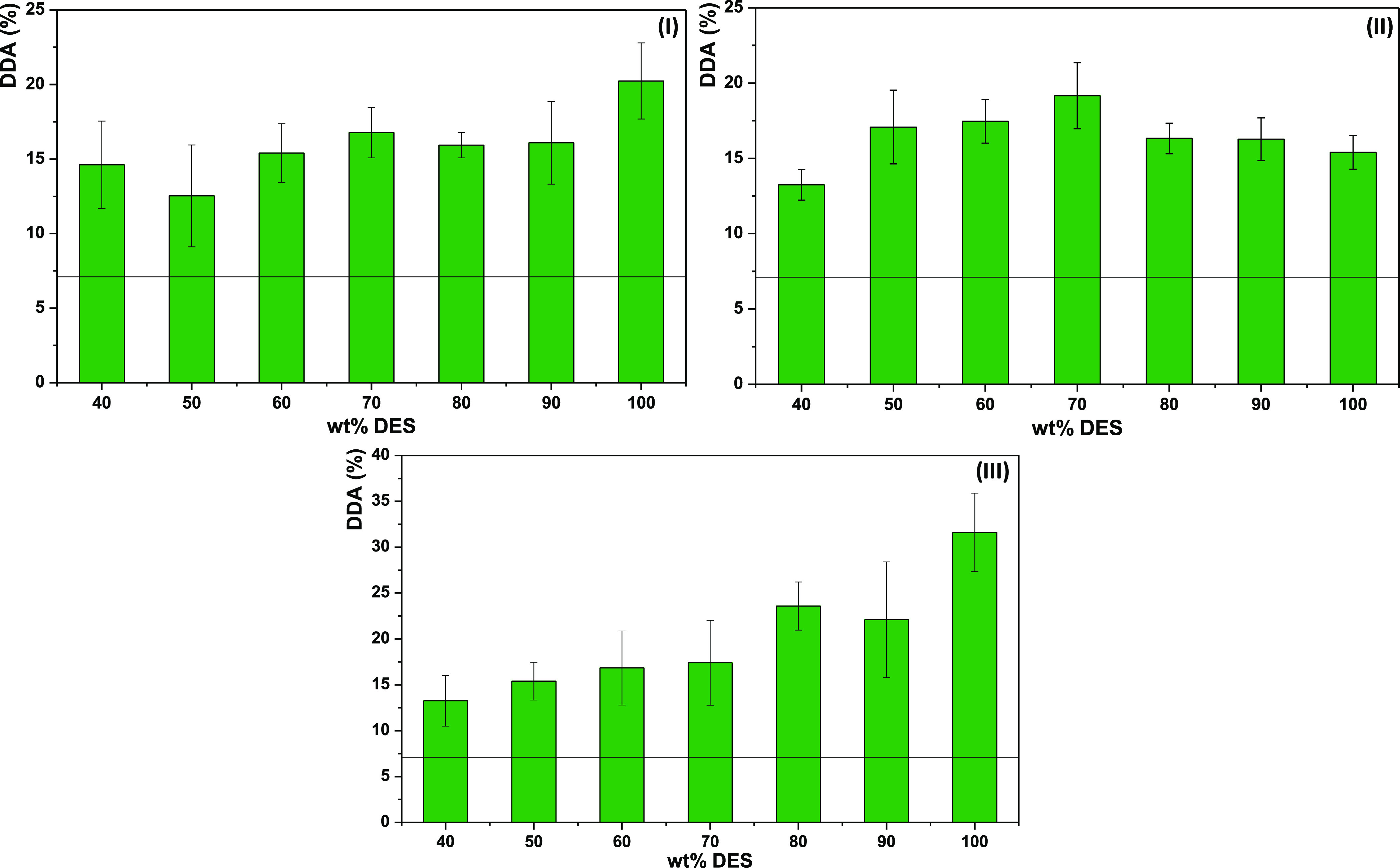
Hydrotropy study for the most promising DESs upon chitin deacetylation at 80 °C for 24 h: (I) K_2_CO_3_:G, (II) [Ch]Cl:AA, and (III) [Ch]Cl:MA. The line represents the DDA of commercial chitin (7 ± 1%).

In this sense, a hydrotropy study was performed for the most promising DESs, namely, K_2_CO_3_:G and [Ch]Cl:MA, and for [Ch]Cl:AA owing to the high fluidity of the system, which can facilitate the deacetylation reaction. The results shown in Figure 5 demonstrate that the DESs follow two different patterns. When deacetylation is carried out by K_2_CO_3_:G and [Ch]Cl:MA, the DDA increases with the amount of DES present in the solution, demonstrating a monotonic increase of chitin solubility and deacetylation. In these cases, a pure DES is required for a more efficient deacetylation. In contrast, when [Ch]Cl:AA was applied for the deacetylation, there was a nonmonotonic solubility and deacetylation enhancement with the DES concentration, with a maximum at intermediate DES concentrations. This indicates that [Ch]Cl:AA is able to act as a hydrotrope. Herein, solute–hydrotrope (chitin–[Ch]Cl:AA) interactions are established between their apolar moieties, resulting in strong and favorable interactions only due to the presence of water.^48^ Consequently, at 70 wt %, there is a higher solubilization and subsequent deacetylation of chitin, which represents an approximate 5% increase in the DDA in comparison with the pure DES. However, since chitin is a long-chain polymer, it is difficult for this hydrotrope to fully form “aggregates” that allow not only its solubilization but also deacetylation.

Overall, these results show that K_2_CO_3_:G and [Ch]Cl:MA allow the solubilization of chitin through the typically observed cosolvency mechanism, whereas [Ch]Cl:AA displays a hydrotropic mechanism. Independent of the subjacent solubilization mechanism and at the best conditions so far, K_2_CO_3_:G, [Ch]Cl:AA, and [Ch]Cl:MA led to a DDA of 20 ± 3, 19 ± 2, and 32 ± 4%, respectively. This represents already a noteworthy modification of the chitin structure, especially considering that almost no solvents are able to solubilize chitin.

#### Influence of HBA, HBD, and DES on Chitin Deacetylation

When DESs are applied in reaction, extraction, and separation processes, it is important to also evaluate how the HBA and HBD perform *per se*. This is of special relevance when aqueous solutions of DESs are involved as, depending on the amount of water present, the DES might no longer be present as a DES but instead be an aqueous solution of two species. To pinpoint the additional *mechanistic* effect of DES on deacetylation beyond simple solubility mediation, first-principles calculations were employed. In short, the calculated barriers for deacetylation with different DES formulations should be lower than those of their separated constituent parts (*e.g.*, simple alcohols, acids, or [Ch]Cl).

Experimentally, we have shown that deacetylation proceeds in several DESs of different formulations: [Ch]Cl:alcohol, [Ch]Cl:organic acid, and K_2_CO_3_:G. We therefore investigate the mechanisms when only single components are present and compare the calculated barriers with those for complex DESs (see the section Computational Screening).

##### Alcohols

The single-component mechanism can follow a one-step or a two-step mechanism. In a one-step mechanism, the oxygen of the hydroxyl group binds to the amidic carbon, while the hydroxyl hydrogen moves to amidic nitrogen. The amidic C–N bond is cleaved simultaneously. This barrier is 40 kcal·mol^–1^. In a two-step mechanism, upon the formation of the C–O bond, the hydroxyl hydrogen binds to the same oxygen atom. In the subsequent step, hydrogen migrates to the amidic nitrogen, causing a C–N bond cleavage. The barriers for these steps are 31 and 27 kcal·mol^–1^. For glycol, the calculated barriers are consistently slightly higher, *i.e.*, 42 kcal·mol^–1^ for the one-step mechanism and 34 and 3 kcal·mol^–1^ for the two-step mechanism.

##### Choline Chloride

In pure choline chloride, which is an alcohol, the reaction *could* follow a similar mechanism. However, the barriers are 54 kcal·mol^–1^ (single-step mechanism) or 44 and 47 kcal·mol^–1^ (two-step mechanism), making the reaction unlikely. Choline chloride on its own is thus not conducive to the reaction. As stated before, the calculated barrier for [Ch]Cl:G was 31 kcal·mol^–1^, showing that it is glycerol that is the most responsible for deacetylation.

##### Organic Acids

**Figure 6:**
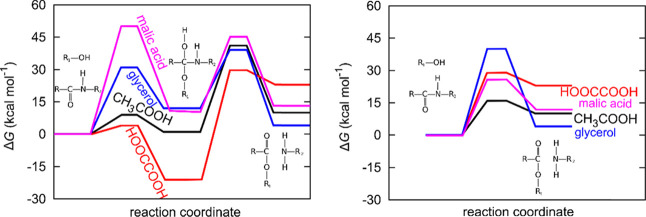
Calculated Gibbs free energies for the GlcNAc deacetylation with glycerol, acetic, oxalic, and malic acids in a two-step mechanism (I) and one-step mechanism (II).

When using acetic, oxalic, or malic acid, there are two possible routes. In concentrated aqueous solutions of suitably strong bases, a sufficient concentration of H_3_O^+^ enables the previously mentioned mechanism (H_3_O^+^-mediated reaction). However, even with no appreciable H_3_O^+^ concentration (either being weak or in a nonaqueous medium), acids can catalyze the deacetylation. In a one-step mechanism, the carboxylic oxygen atom attaches to the amide carbon atom, while the carboxylic hydrogen atom (on the other oxygen atom) binds to the nitrogen atom, cleaving the C–N bond in the process. The Gibbs barriers for this process are 16, 29, and 24 kcal·mol^–1^ for acetic, oxalic, and malic acids, respectively. In a two-step mechanism, the carboxylic oxygen again binds to the amide carbon atom, but the carboxylic hydrogen protonates the amide oxygen atom instead. The ensuing intermediate breaks apart through a proton migration from O to N. The barriers for these steps are 9 and 30 kcal·mol^–1^ for acetic acid, 10 and 31 kcal·mol^–1^ for oxalic acid, and 51 and 41 kcal·mol^–1^ for malic acid. Figure 6 depicts the corresponding Gibbs free energy of one-step and two-step mechanisms.

The results are consistent with experimental data, as well. The barrier for pure AA is lower than for [Ch]Cl:AA, and [Ch]Cl:AA performs the worst among the three in deacetylation. We argue that any DDA we see in this case is due to the residual effect of AA and not of the DES interplay. For oxalic and malic acids, the barriers are higher than in their combination with ChCl (29 vs 26 kcal·mol^–1^ for AA, and 25 vs 24 kcal·mol^–1^ for OA). These two formulations ([Ch]Cl:MA and [Ch]Cl:OA) exhibited the best results.

**Figure 7:**
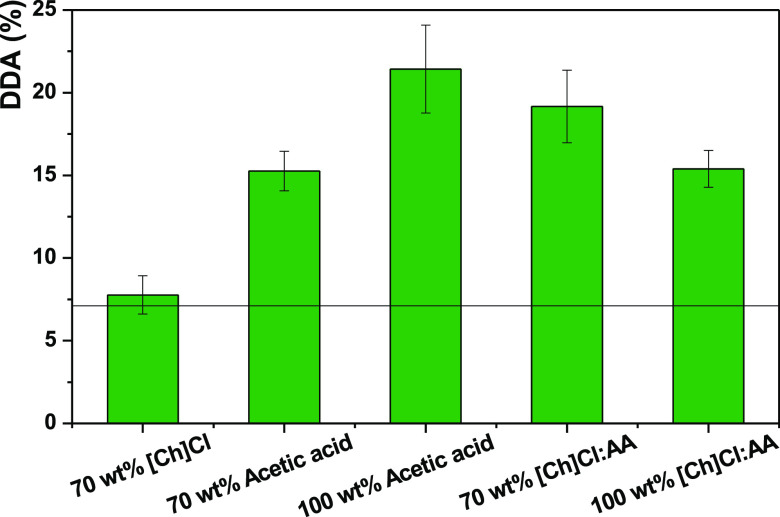
Influence of the HBA ([Ch]Cl), HBD (acetic acid), and DES ([Ch]Cl:AA) on DDA at 80 °C for 24 h. The line represents the DDA of commercial chitin (7 ± 1%).

Experimentally, this effect was studied on [Ch]Cl:AA for two main reasons: (i) this is the only DES with a hydrotropic mechanism and (ii) AA is liquid (MA and OA are solid), allowing the study of a pure solvent. It was previously seen that 70 wt % [Ch]Cl:AA led to the highest deacetylation. Hence, aqueous solutions of the HBA ([Ch]Cl) and HBD (acetic acid) of this concentration were prepared for comparison with the respective DES. Pure components were also tested. The results are shown in Figure 7. When the aqueous solutions are considered, it is evident that the DDA follows the trend HBA < HBD < DES. Pure [Ch]Cl does not promote deacetylation (above the initial 7%), which is also consistent with DFT calculations (the rate-determining barrier of 44 kcal·mol^–1^ is too high). In contrast, acetic acid (in an aqueous solution of 70 wt %) can react with chitin, up to some extent, resulting in a DDA of ∼15% (barrier of 16 kcal·mol^–1^). The efficiency is enhanced when the HBA and HBD are combined, reaching ∼20% DDA. This is a complex effect, caused by the structure of chitin, and is therefore not captured by the DFT calculations (which were performed on GlcNAc and show a *larger* barrier of 26 kcal·mol^–1^). This corroborates the existence of the synergistic effect of the DES components, as previously observed in different works.^49,50^

When pure solvents are concerned, however, it is evident that acetic acid and the respective DES have different solubilization mechanisms that consequently lead to a distinct deacetylation efficiency. Pure acetic acid exhibits the cosolvency mechanism (DDA increases with concentration), whereas [Ch]Cl:AA displays a hydrotropic mechanism as mentioned before. Consequently, the availability of the acetic acid and its interaction with the solute seem to be more efficient than for the DES as it promotes a higher degree of deacetylation.

#### Temperature and Reaction Time Influence on Chitin Deacetylation

**Figure 8:**
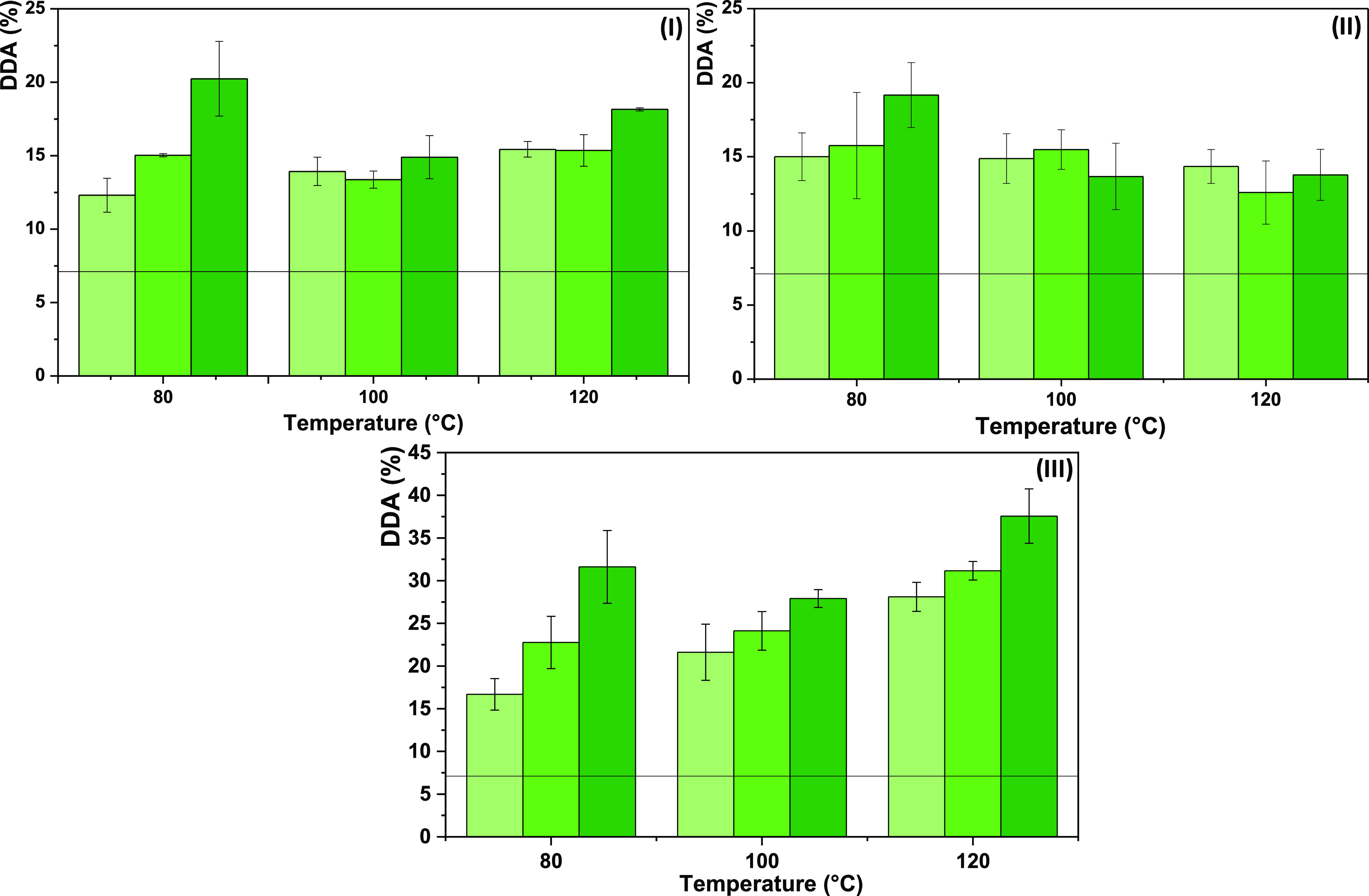
Influence of time (light-green solid box, 4 h; green solid box, 6 h; dark-green solid box, 24 h) and temperature (80, 100, and 120 °C) on chitin deacetylation for the most promising DESs: (I) K_2_CO_3_:G, (II) 70 wt % [Ch]Cl:AA, and (III) [Ch]Cl:MA. The line represents the DDA of commercial chitin (7 ± 1%).

As temperature and time are two of the most crucial parameters in organic transformations and reactions, their influence on chitin deacetylation was investigated to optimize the protocol. On K_2_CO_3_:G, 70 wt % [Ch]Cl:AA, and [Ch]Cl:MA, the temperature was varied between 80 and 120°C and shorter time ranges were tested and compared with the initial 24 h deacetylation. Figure 8 depicts the results.

K_2_CO_3_:G and [Ch]Cl:AA both achieve an ∼20% DDA at 80 °C after 24 h. The reaction is faster with the latter, with DDA reaching ∼15% after 4 h, while in K_2_CO_3_:G the reaction is slower. At higher temperatures, the reaction is faster (the difference between DDA after 4 h and 24 h decreases). However, increasing the temperature *decreases* DDA, the effect being more pronounced for [Ch]Cl:AA, where DDA tends to stabilize around 15%. At 100 and 120 °C, the difference between DDA after 4, 6, and 24 h is not statistically relevant. In contrast, [Ch]Cl:MA exhibits much more clearcut effects. We observe an increase in DDA with reaction time. The temperature effect is less pronounced, but increasing it to 120 °C still improved the DDA to ∼40%. Furthermore, in one run, the reaction time was extended to 96 h, as shown in Figure S8. No further increase in DDA was observed.

In summary, by properly choosing the DESs, it is possible to optimize a green chitin deacetylation with a DDA up to at least ∼40%. Efforts in further improving the DDA were not successful. We believe that this is due to the fact that the biopolymer deacetylation should be mainly occurring in the amorphous parts of chitin. Moreover, chitin is obtained after precipitation with water, resulting in an aqueous solution of DES. Upon ultrafiltration to separate the acetate, the secondary product of this reaction, it is possible to evaporate the water and reuse the DES, as it has been recently shown for similar DESs.^51^

#### Chitin Deacetylation with NaOH Mineral Base

**Figure 9:**
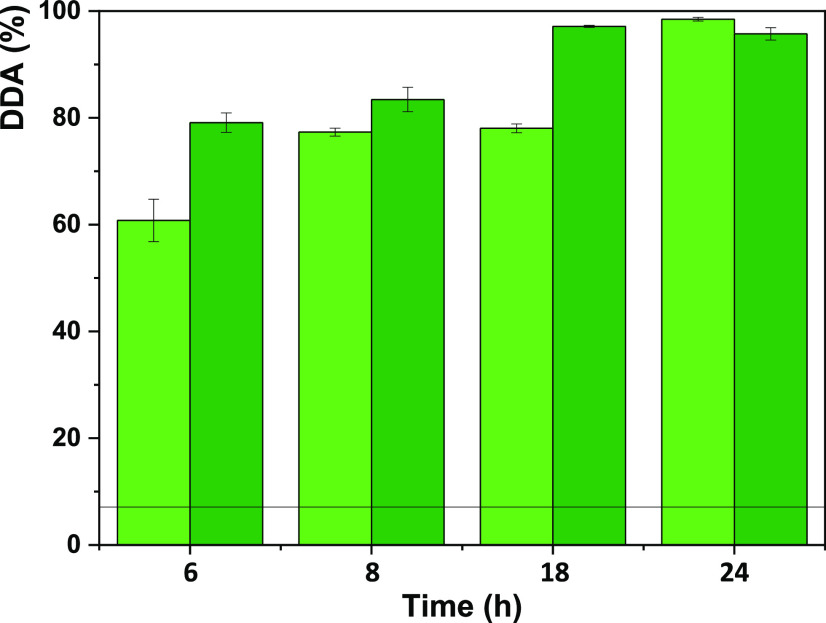
Influence of time and NaOH concentration upon chitin deacetylation at 80 °C: Green solid box, 40 wt % NaOH; dark-green solid box, 50 wt % NaOH. The line represents the DDA of commercial chitin (7 ± 1%).

Lastly, different NaOH concentrations (40 and 50 wt %) were used to perform the conventional chitin deacetylation reported in the literature. This allows a direct comparison between the alternative deacetylation process (DES) and the typical one applied. This is necessary to compare the efficiency of DESs in a controlled fashion as it is well-known that chitin derived from different species as well as the DDA determination through distinct techniques lead to very different results. Herein, the influence of different NaOH concentrations was studied over time, as shown in Figure 9.

The results demonstrate that 6 h suffice for an efficient conversion of chitin into chitosan and that the DDA obtained ranges between 60 and 98%. When 40 wt % NaOH was used, at least 8 h was required to obtain a high DDA (≥80%). However, when using 50 wt %, 6 h suffice for comparable DDA.

When performing deacetylation in a real setting, several factors should be considered, especially what DDA is required for a specific task. This allows for an informed evaluation if further processing is required and what is the most feasible economic and sustainable option: using lower alkaline conditions for longer periods or increasing the NaOH concentration but reducing the reaction time. The latter is especially crucial when a fully deacetylated chitosan is required since there is a 6 h reduction of the reaction duration (24 vs 18 h) when 50 wt % NaOH instead of 40 wt % is used.

This data is comparable to that in the literature. A detailed comparison is less informative due to the variation of parameters among studies: source of chitin, deacetylation method, analytical technique, NaOH concentration, duration of the reaction, temperature under study, and the solid/liquid ratio used.^52−55^ Singh et al.^52^ also used 50 wt % NaOH and a 1:50 (w/v) solid/liquid ratio to study chitin deacetylation but performed it at higher temperatures (110 and 130 °C) and for shorter periods of time (2–8 h). DDA was also analyzed using FTIR and while applying the exact same equation here reported. As expected, their results showed an increasing DDA trend with temperature and the duration of the reaction, which is consistent with our data. Interestingly, after 8 h of deacetylation and while using the same NaOH concentration, we achieved a DDA of 83 ± 2% at 80 °C, whereas Singh et al. reported a DDA of 86.8 ± 0.4% only at 110°C.^52^ This evidences that the chitin source and the deacetylation method play a major role in the process. Galed and co-workers^53^ showed that it was possible to achieve a DDA of ∼80% in ca. 1 h when using extremely concentrated NaOH (70 wt %) and high temperatures (110 °C). However, with 50 wt % NaOH at 110 °C, they only achieved a DDA of approximately 70% even after 5 h.

Comparing chitin deacetylation using DES or NaOH, it was obvious that even the lowest tested NaOH concentration (40 wt %) after 6 h outperforms the optimized DES protocol ([Ch]Cl:MA, 24 h, 120°C) with a DDA of 60% compared to 40%.

Chitin is only considered chitosan upon the deacetylation of 50% of its amino groups; hence, this biocompatible deacetylation of chitin is merely 10% shy of the threshold. In terms of sustainability, this work reports a much greener process that is still able to promote a noteworthy deacetylation and increase chitin reactivity. As this is the first successful application of DES for chitin deacetylation, representing by no means an exhaustive screening campaign, we believe that the developed protocol will be further improved in the future. The final product with 40% of the *N*-acetyl groups present removed is more prone to further processing. Lastly, with a 40% DDA, the range of possible applications of chitin vastly expands.^55^

#### XRD

**Figure 10:**
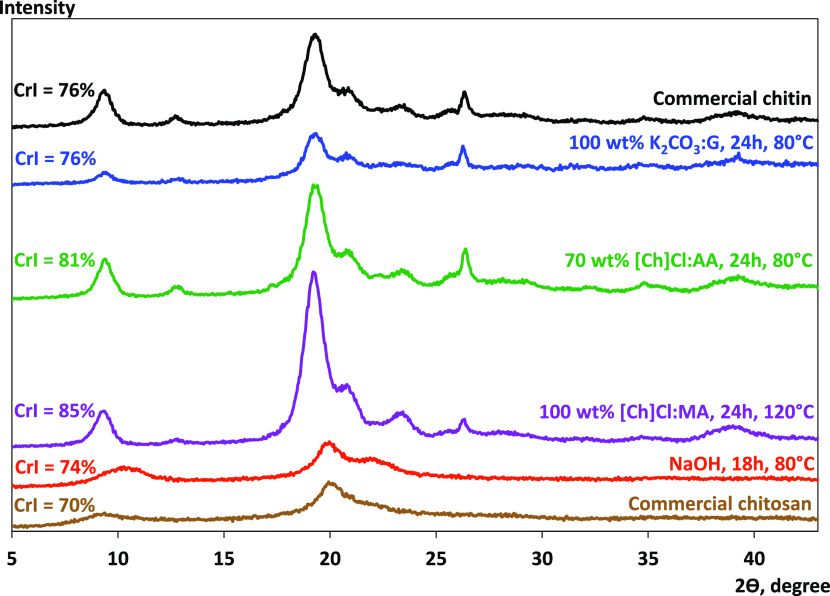
X-ray diffraction patterns and crystallinity index (CrI) of commercial chitin and chitosan as well as the samples obtained with the most promising DESs studied in this work: 100 wt % K_2_CO_3_-G and 70 wt % [Ch]Cl:AA after 24 h of deacetylation at 80 °C and 100 wt % [Ch]Cl:MA after 24 h of deacetylation at 120 °C and the sample obtained with 50 wt % NaOH after 18 h of deacetylation at 80 °C.

XRD patterns of commercial chitin and chitosan are displayed in Figure 10 as well as the crystalline peaks from the samples obtained with the most promising DESs studied in this work: 100 wt % K_2_CO_3_-G and 70 wt % [Ch]Cl:AA after 24 h of deacetylation at 80 °C and 100 wt % [Ch]Cl:MA after 24 h of deacetylation at 120°C. Chitosan obtained using 50 wt % NaOH after 18 h of deacetylation at 80 °C was also analyzed for comparison purposes. Chitin displays two characteristic crystalline reflections at 2θ ≈ 9.4 and 19.3°, whereas chitosan presents its crystalline reflections at 2θ ≈ 9–10 and 20°. This data is in agreement with that in the literature.^17,56,57^ The peak at 2θ ≈ 9–11° corresponds to amide I (−N–CO–CH_3_), while the peak at 2θ ≈ 19–20° corresponds to amide II (−NH_2_).^58^ Regarding the results obtained with DES, it is evident that all of the systems present an identical spectrum to chitin, which was expected considering the results attained with FTIR. Additionally, these samples presented a crystallinity index identical to or higher than that of chitin. This was not entirely anticipated since typically the crystallinity index decreases with the DDA increase^59^ and with methods affecting the surface of the polymer.^57^ However, there has been one study that has reported the crystallinity index to have increased alongside the DDA increase.^58^ Herein, the chitosan sample with the lowest DDA of ∼80% presented a crystallinity index of nearly 63% and showed an increasing trend up to a crystallinity index of ∼71% when the DDA was higher than 95%. The crystallinity index is always dependent on the source and type of chitin, and it can be correlated, to some extent, with the DDA and the molecular weight of the final product.^58,59^ When the deacetylation was carried out with 50 wt % NaOH, the chitosan formed displays a regular crystalline pattern as the commercial chitosan, with a crystallinity index lower than that of chitin.

## Conclusions

This works reports on the previously unreported ability of DES to promote chitin deacetylation. A combined DFT theoretical and experimental study revealed the most effective DES for deacetylation and optimized conditions.

First, quantum chemical calculations for the deacetylation of GlcNAc with several reagents were performed. Calculating the barriers for alkaline or acidic hydrolysis, which are known to proceed, as a benchmark, we have screened the most common DESs for deacetylation. K_2_CO_3_:G, [Ch]Cl:OA, and [Ch]Cl:MA were recognized as the most promising. The mechanism of deacetylation in K_2_CO_3_:alcohol proceeds in a two-step fashion: first the carbonate ion binds to the amidic carbon with a barrier of 26 kcal·mol^–1^ and then the C–N bond is cleaved. In [Ch]Cl:acid mixtures, the concerted and two-step mechanisms are both possible with barriers of 20–25 kcal·mol^–1^. Malic and oxalic acid performed the best. In [Ch]Cl:alcohol mixtures, however, the deacetylation is not facilitated compared to alcohols.

Based on DFT screening, experimental tests were performed. In total, 10 different DESs were screened, and K_2_CO_3_:G, [Ch]Cl:AA, and [Ch]Cl:MA showed the most promising results with initial DDA ∼20% at 80 °C after 24 h. These three were further investigated to tweak the protocol with respect to concentration, temperature, and time. [Ch]Cl:OA, although performing well in shorter timeframes, was not evaluated further due to decomposition of the initial polymer and physical unsuitableness of the product.

To experimentally verify the mechanism of solubilization, the effect of DES concentration was tested for K_2_CO_3_:G, [Ch]Cl:MA, and [Ch]Cl:AA. On the one hand, K_2_CO_3_:G and [Ch]Cl:MA displayed a monotonic increase in chitin solubility and DDA with an increase in the DES concentration. In pure solvents, DDA was the highest. On the other hand, [Ch]Cl:AA presented a nonmonotonic solubility dependence and deacetylation enhancement upon the DES concentration, with a maximum at intermediate DES concentrations (70 wt %). This indicates that [Ch]Cl:AA is able to act as a hydrotrope.

Upon varying the reaction parameter, the highest achieved DDA was 40% when using [Ch]Cl:MA for 24 h at 120 °C. Although this is still shy of the 50% threshold, conventionally proposed as a delineation between chitin and chitosan, the DESs were able to considerably increase chitin’s reactivity, thus making it more prone to further dissolution and processing. We stress that there are countless possible combinations of DESs. Although this screening campaigning 10 possibilities falls short of the threshold, it still represents the first successful application of DESs for chitin deacetylation. Further studies on DES-mediated biocompatible and sustainable deacetylation of chitin will undoubtedly improve this value considerably.

To sum up, in this work, we demonstrated a novel method for the deacetylation of chitin, achieving considerable conversions. This approaches a full-fledged transformation of chitin into chitosan while using solely biocompatible solvents, which has not, to the best of our knowledge, been previously reported. By removing a substantial amount of *N*-acetyl groups from chitin, its reactivity increased and made polymers more accessible for additional processing. This further increases the range of chitin’s application, for instance, in technical and agricultural applications.^55^

## Supporting Information Available

Influence of reaction time on chitin deacetylation while using pure [Ch]Cl:OA and K_2_CO_3_:G (PDF)
