# Supplementary material for: Chitin Deacetylation Using Deep Eutectic Solvents: Ab Initio-Supported Process Optimization
Source: ACS Sustain Chem Eng. 2021 Mar 5;9(10):3874–86. doi: 10.1021/acssuschemeng.0c08976 (PMC8025712; doi:10.1021/acssuschemeng.0c08976)
Supplement: Supplementary file 1 — sc0c08976_si_001.pdf [file sc0c08976_si_001.pdf]

Chitin Deacetylation Using Deep Eutectic Solvents: *Ab Initio*-Supported Process Optimization

Filipa A. Vicente,[†,*] Matej Huš, [†,‡] Blaž Likozar,[†] Uroš Novak[†,*]

[†]Department of Catalysis and Chemical Reaction Engineering, National Institute of Chemistry, Ljubljana, Slovenia

[‡]Association For Technical Culture of Slovenia (ZOTKS), Zaloška 65, 1000 Ljubljana, Slovenia.

*Corresponding authors: filipa.andre.vicente@ki.si; uros.novak@ki.si

Supporting Information Contents:

Number of pages: 6

Number of Figures: 8

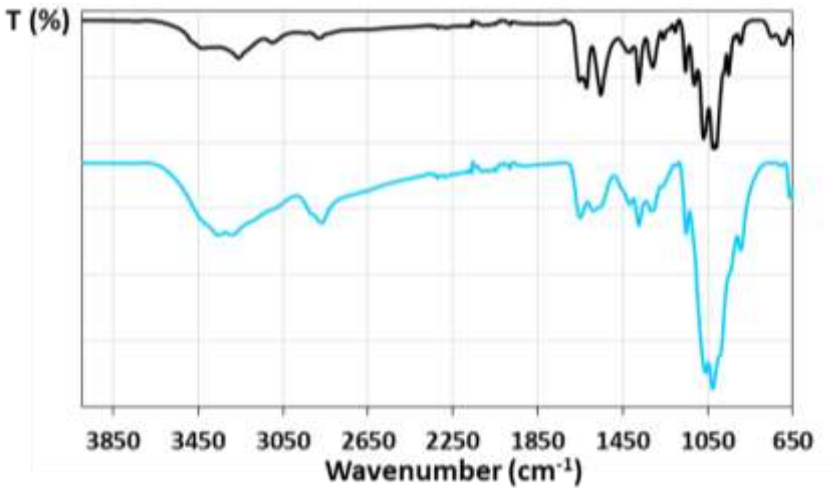

Figure S1. FTIR spectra of the commercial chitin (---) and chitosan (---).

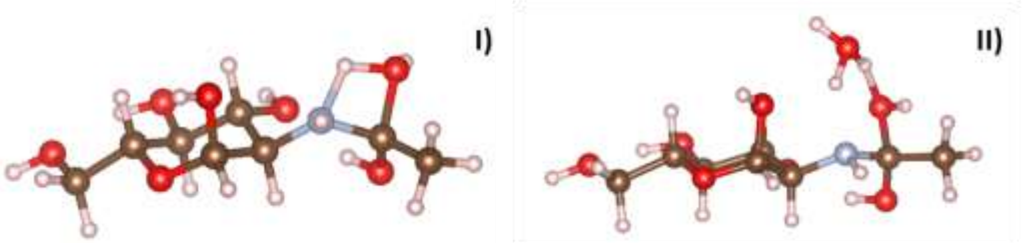

Figure S2. Two possible transition states for the additional of water to the protonated amide in acidic water solutions with (I) one and (II) two water molecules participating.

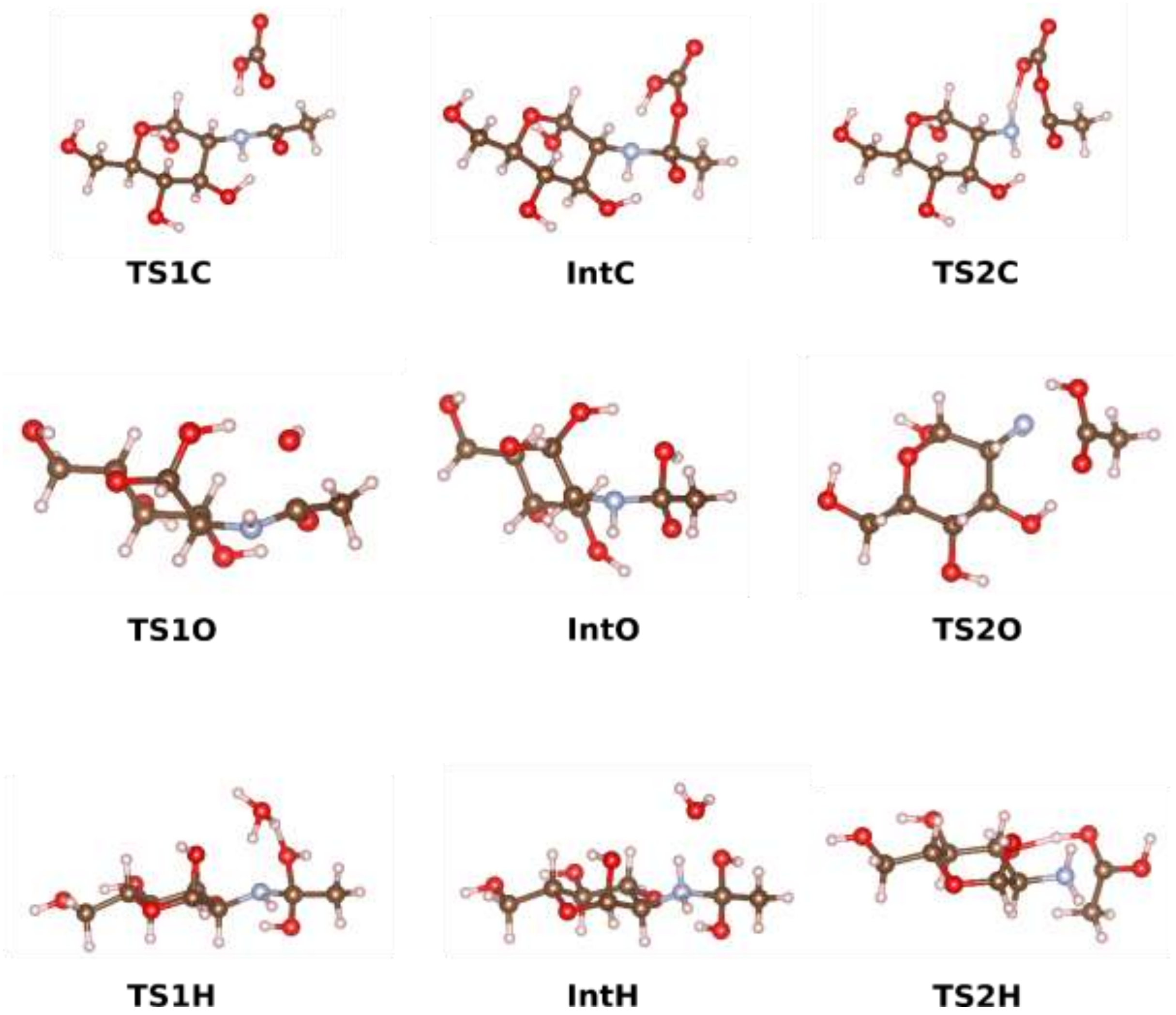

Figure S3. Structures of intermediates and transition states in deacetylation of GlcNAc with H_3O^+, OH^- and HCO_3^- for the potential energy surface in **Figure 2**.

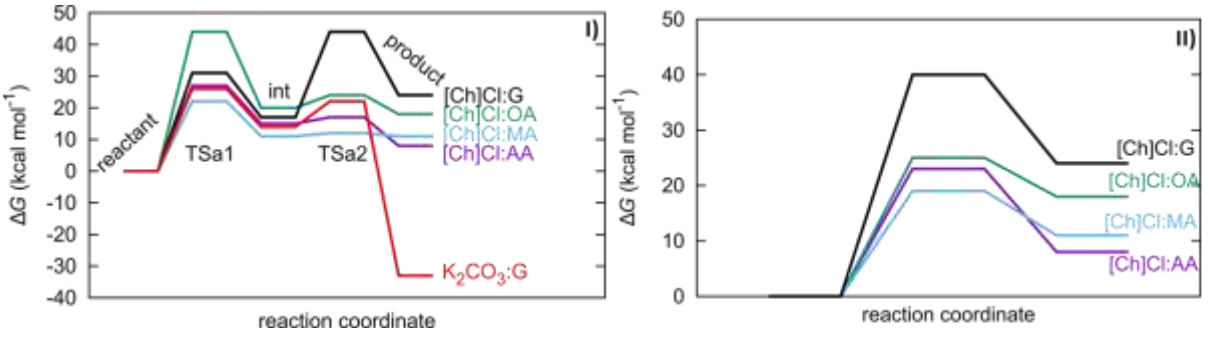

Figure S4. The calculated Gibbs free energies for amide hydrolysis with various DES (I), with a close-up in II).

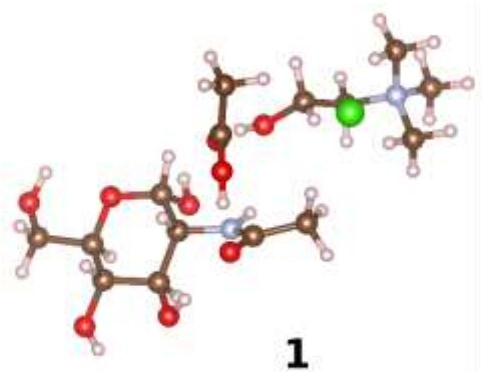

1

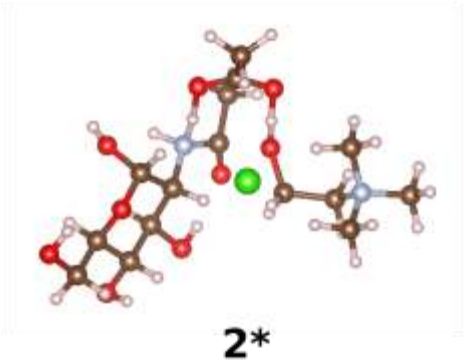

2*

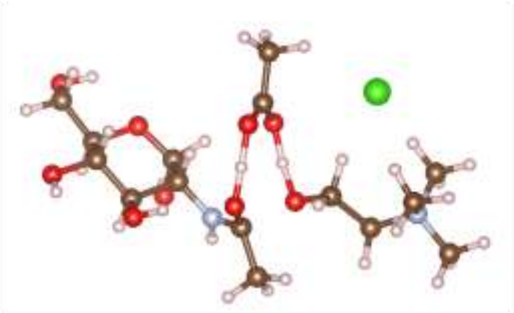

3*

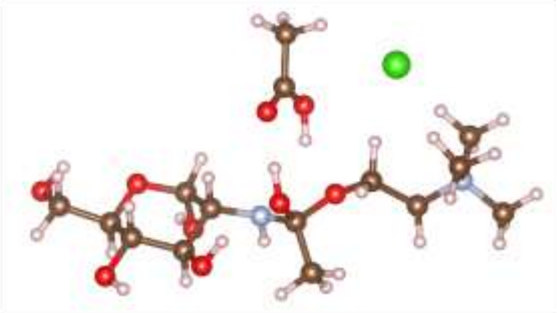

4

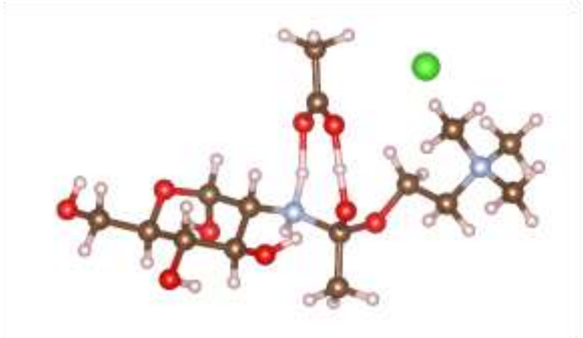

5*

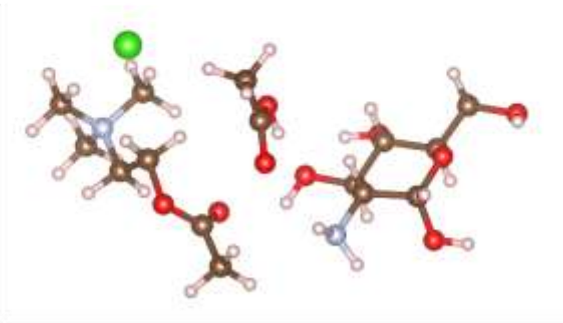

6

Figure S5. Structures of intermediates and transition states in deacetylation of GlcNAc with [Ch]Cl:AA, for which skeletal formulae are shown in **Figure 3**.

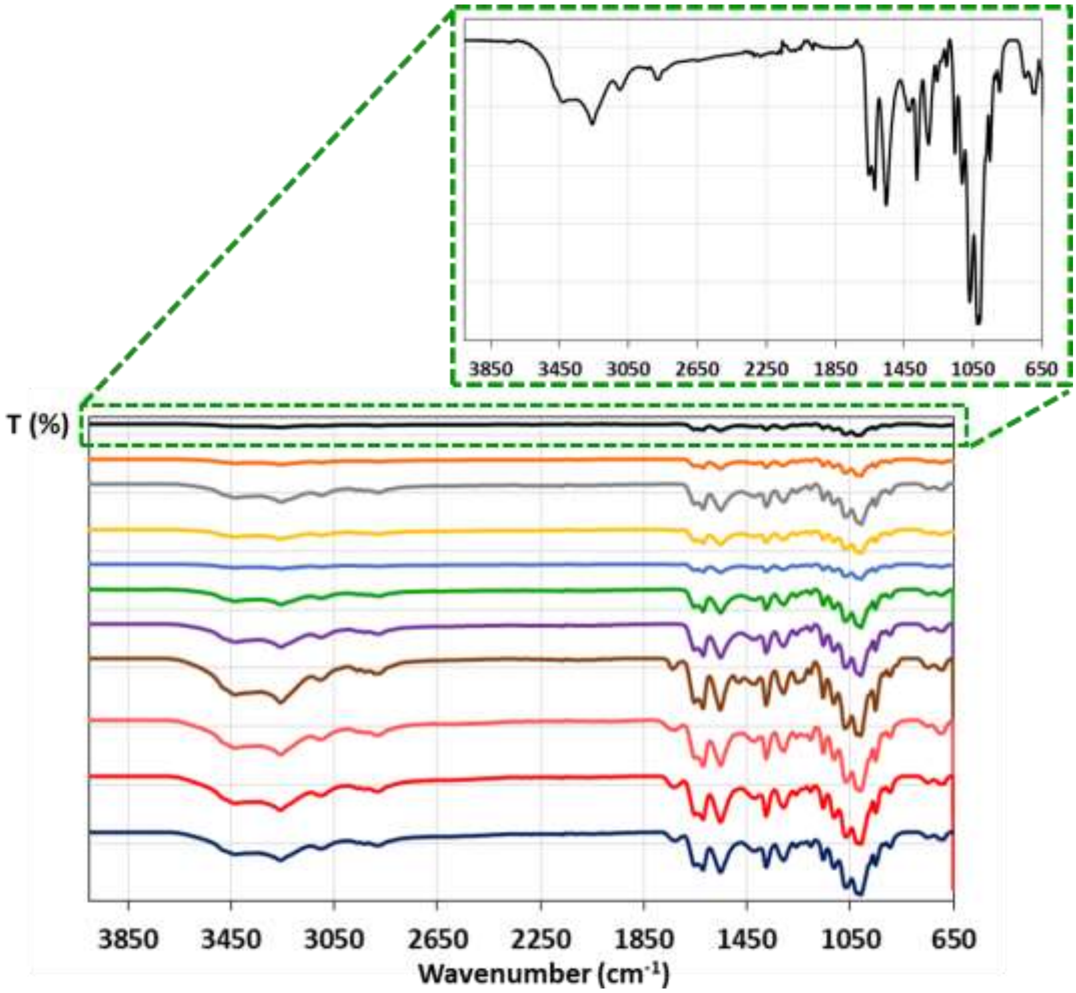

Figure S6. FTIR spectra of the DES screening upon its ability to perform chitin deacetylation at 80°C for 24h: **---**, commercial chitin; ---, $KHCO_3$:G; ---, K_2CO_3:G; ---, [Ch]DHC:G (1:2); ---, [Ch]DHC:G (1:4); ---, [Ch]Cl:G; ---, [Ch]Cl:EG; ---, [Ch]Cl:AA; ---, [Ch]Cl:OA; ---, [Ch]Cl:MA; ---, [Ch]Cl:CA. In the particular case of [Ch]Cl:OA, the deacetylation was performed at 80°C but for 4h.

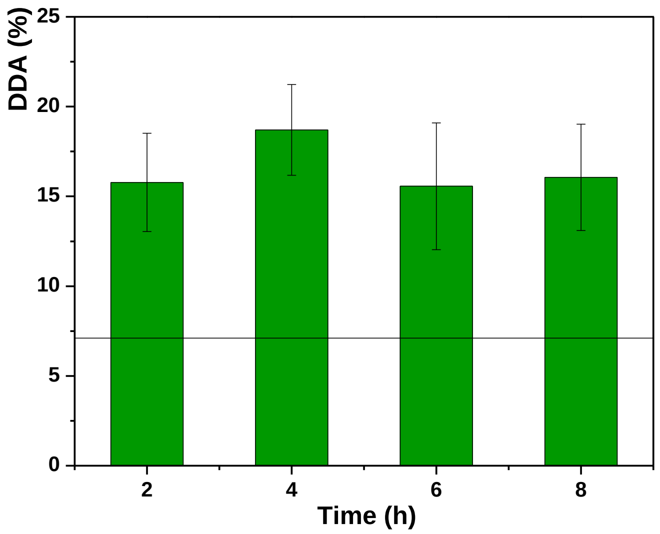

Figure S7. Influence of the reaction time on chitin deacetylation while using pure [Ch]Cl:OA at 80°C. Line represents the DDA of commercial chitin (7 ± 1%).

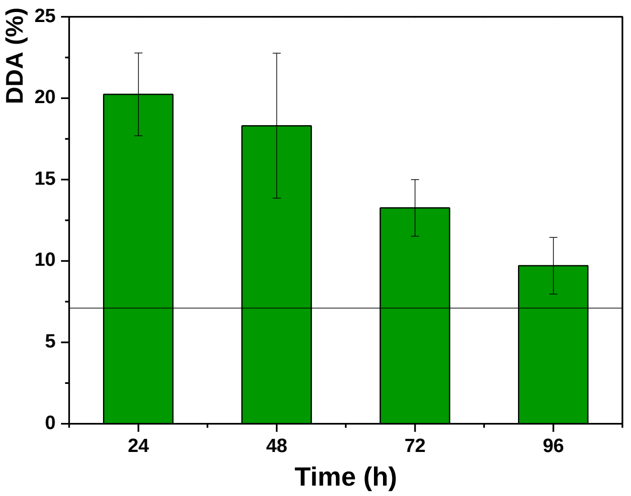

Figure S8. Influence of the reaction time on chitin deacetylation while using pure K_2CO_3-G at 80°C. Line represents the DDA of commercial chitin (7 ± 1%).
